# Living with chronic illness scale: international validation of a new self-report measure in Parkinson’s disease

**DOI:** 10.1038/npjparkd.2016.22

**Published:** 2016-10-20

**Authors:** Leire Ambrosio, Mari Carmen Portillo, Carmen Rodríguez-Blázquez, Mayela Rodriguez-Violante, Juan Carlos Martínez Castrillo, Víctor Campos Arillo, Nélida Susana Garretto, Tomoko Arakaki, Marcos Serrano Dueñas, Mario Álvarez, Ivonne Pedroso Ibáñez, Ana Carvajal, Pablo Martínez-Martín

**Affiliations:** 1 Faculty of Nursing, University of Navarre, C/ Irunlarrea, s/n, Edificio de los Castaños, Pamplona, Spain; 2 Faculty of Health Sciences, University of Southampton, Southampton, UK; 3 Area of Applied Epidemiology, National Centre of Epidemiology and CIBERNED, Carlos III Institute of Health. Av. Monforte de Lemos, Madrid, Spain; 4 Movement Disorders Unit, National Institute of Neurology and Neurosurgery, Movement Disorders Clinic, Mexico City, Mexico; 5 Neurology Unit, Ramon y Cajal University Hospital, IRYCIS, Ctra, de Colmenar Viejo, Madrid, Spain; 6 Neuroscience Area, Vithas-Xanit International Hospital, Avenida de los Argonautas s/n, Benalmádena, Spain; 7 Department of Neurology, JM Ramos Mejia Hospital, Buenos Aires, Argentina; 8 Movement Disorder and Biostatistics Units, Neurological Service, Carlos Andrade Marín Hospital, Quito, Ecuador; 9 Department of Movement Disorders and Neurodegeneration, CIREN, La Habana, Cuba

## Abstract

Understanding how a person lives with a chronic illness, such as Parkinson’s disease (PD), is necessary to provide individualized care and professionals role in person-centered care at clinical and community levels is paramount. The present study was aimed to analyze the psychometric properties of the Living with Chronic Illness-PD Scale (EC-PC) in a wide Spanish-speaking population with PD. International cross-sectional study with retest was carried out with 324 patients from four Latin American countries and Spain. Feasibility, acceptability, scaling assumptions, reliability, precision, and construct validity were tested. The study included 324 patients, with age (mean±s.d.) 66.67±10.68 years. None of the EC-PC items had missing values and all acceptability parameters fulfilled the standard criteria. Around two-third of the items (61.54%) met scaling assumptions standards. Concerning internal consistency, Cronbach’s alpha values were 0.68–0.88; item-total correlation was >0.30, except for two items; item homogeneity index was >0.30, and inter-item correlation values 0.14–0.76. Intraclass correlation coefficient for EC-PC stability was 0.76 and standard error of measurement (s.e.m.) for precision was 8.60 (for a EC-PC s.d.=18.57). EC-PC presented strong correlation with social support (*r*_S_=0.61) and moderate correlation with life satisfaction (*r*_S_=0.46). Weak and negligible correlations were found with the other scales. Internal validity correlations ranged from 0.46 to 0.78. EC-PC total scores were significantly different for each severity level based on Hoehn and Yahr and Clinical Impression of Severity Index, but not for Patient Global Impression of Severity. The EC-PC has satisfactory acceptability, reliability, precision, and validity to evaluate living with PD.

## Introduction

Demographic changes happening in the twentieth century in the Western world such as ageing and increase in life expectancy have produced a significant growth in chronic illnesses in the contemporary society. In particular, chronic diseases have become a leading health-related issue.^1^

Among chronic diseases, neurodegenerative and progressive disorders such as Parkinson’s Disease (PD) stand out. PD is the second most common neurodegenerative disease, after Alzheimer’ disease, affecting 1% of all people over 60 years of age in industrialized countries.^2^ PD prevalence is increasing and it is expected that the number of PD patients will double by 2030.^3^ PD is a complex and disabling disorder manifested through a combination of characteristic motor signs, such as bradykinesia, rigidity, resting tremor, and postural instability and non-motor symptoms such as, for example, psychiatric disorders, autonomic disturbances, pain, and fatigue.^4^ Throughout the PD course, patients experience a progressive intensification of symptoms and increasing limitations to the performance of daily activities.

Living with a chronic illness, such as PD, is a complex, dynamic, cyclic, and multidimensional process that involves such components as Acceptance, Coping, Self-management, Integration, and Adjustment.^5^ Several studies show that living with a chronic illness, as PD does not only affect the patients’ physical state but also other aspects in their lives, such as the emotional and social ones.^5–7^ In this context, healthcare professionals need to adopt an integral approach so that all aspects of the person, understood as a bio-psychosocial and spiritual being, are addressed.^6–8^ In particular, clinical specialists have an essential role in facilitating the patient’s living with PD process and, consequently, improving his/her quality of life and well-being.^7,8^ It is, therefore, important to have available tools for assessing living with PD from the patient’s perspective, in combination with clinical tools that focus on specific signs and disabilities of PD. Nowadays, despite the great number of existing measures, there are no tools to evaluate the process of living with a chronic illness, such as PD. In particular, the existing tools evaluate the process in a fragmented way. For example, the Scales for Outcomes in Parkinson’s Disease-Psychosocial (SCOPA-PS)^9^ only evaluates the psychosocial adjustment to PD without bearing in mind other components of the daily living with the disease such as Acceptance or Self-management, for example. In this way, the Living with Chronic Illness-PD Scale (EC-PC, from Spanish ‘*Escala deConviviencia con un ProcesoCrónico*’) is the only available tool in clinical practice and research to evaluate how the patient is living with PD in a comprehensive manner, focusing on the person and not on the disease. The EC-PC is an innovative self-reported scale, recently developed,^10^ waiting validation studies for testing its psychometric properties. To this purpose, an international collaborative study was carried out in Argentina, Ecuador, Cuba, Mexico, and Spain. The objective of this study was to analyze the psychometric properties of the EC-PC in a wide, multinational Spanish-speaking population.

## Results

Three-hundred twenty-four patients diagnosed with PD, 52.78% men, mean (±s.d.) age 66.67±10.68 (range: 36–94) years were included. In this sample, age at the onset of PD was 56.90±10.92 (range: 24–84) and disease duration 9.76±5.71 (1–28) years. Patients were receiving treatment for PD during 8.82±5.65 (range: 0–28) years and their distribution according the Hoehn and Yahr (HY) stages was: 8.64% in stage 1; 55.25%, stage 2; 30.56%, stage 3; 4.01, stage 4; and 1.54% in stage 5. Two-third (66.36%) were married, 42.90% were retired, and 63.58% had primary or secondary education levels. Table 1 shows the descriptive statistics for disease-related variables and assessments in the study and the EC-PC. Patient-based Global Impression of Severity Scale (PGIS) median (interquartile range) was 3 (2–3).

### Feasibility and acceptability

None of the items had missing values. All acceptability parameters fulfilled the standard criteria. Specifically, floor or ceiling effects were <15% (both, 0.31 for the total score) and skewness values were between −1 and +1 (Table 2).

#### Scaling assumptions

Mean and s.d. values were roughly equivalent, ranging from 1.74 to 3.01 (means) and 1.18 to 1.56 (s.d.). Multitrait-scaling analysis showed that 61.54% of the items had a correct location in their respective domains, whereas the rest of the items (38.46%) performed as “possible errors” in their location.

Cronbach’s alpha was >0.70 for all domains, except for dimension 3-Self-management (alpha=0.68). Two items (12 and 13; 7.69%) did not reach the preset 0.30 threshold value for item-total correlation. Item homogeneity index values were >0.30 for all domains and inter-item correlation coefficient values ranged from 0.14 to 0.76 (Table 2), with only four items (15%, from the domains 2, 3, and 5) under the standard value 0.20.

Test–retest reliability was determined in 55 patients. The ICC for the total scale score was 0.76 and for the dimensions was ⩾0.60, except for the domain 3-Self-management (Table 2). For individual items, weighted kappa ranged from 0.22 (item 13) to 0.80 (item 2).

SEM for EC-PC total score was 8.60 (<½ s.d.: 8.78) and for the dimensions ranged from 1.51 to 2.98, where two of them were <½ s.d. (domain 1-Acceptance and 5-Adjustment; Table 2).

Convergent validity results are shown in Table 3. Correlation coefficient between EC-PC total scores and Duke-UNC Functional Social Support Questionnaire (DUFSS) was 0.61 (strong), whereas correlation between the EC-PC and Satisfaction with Life as a whole was 0.46 (moderate). The EC-PD domain 1-Acceptance showed also moderate correlations with some components of the Satisfaction with Life Scale. EC-PC presented weak correlations with the SCOPA-PS total scale (Table 3). Weak or negligible correlations were found between EC-PC and the clinician-based PD assessments.

Internal validity analysis showed that correlation values between the EC-PC domains ranged from 0.46 to 0.78, except for dimension 1-Acceptance that showed poor correlations with the other dimensions (Table 3).

EC-PC known-groups validity analysis showed that total scores were significantly different for each category of severity (mild, moderate, and severe) based on the HY and CISI-PD, but not for the PGIS (Table 4).

## Discussion

For this first validation study of the EC-PC, carried out in patients with PD, most of the tested psychometric properties resulted in satisfactory values. The patients included in the study (*n*=324) were considered representative of the PD population attending specialized hospital departments or movement disorder units, with over 85% of patients in mild or moderate levels of severity (Table 1). On the other hand, the participation of Spanish-speaking patients from several countries supports the consistency of the results at least for this cultural and linguistic setting.

Concerning the specific validation aspects, EC-PC feasibility and acceptability were satisfactory. Quality of data was excellent, with 100% of cases fully computable, probably due to the close supervision of the participant researchers after completion of the scale by patients. Floor and ceiling effects and the skewness values were into the stipulated range of satisfactory values (Table 2). These findings showed that EC-PC covers the full spectrum of intensity of the construct, with an adequate distribution of the scores suitable to PD population.

Scaling assumptions were deemed acceptable as a whole. The range of means and s.d. distribution were roughly equivalent and 61.54% of the items had a correct location in their corresponding domains. The rest of the items performed in this analysis as “possible errors” in their location, although qualitative data emerged from previous studies in PD^6,7^ support the adequacy of their location. Moreover, other tools for assessment of living with chronic illness components such as Coping and Self-management also include items with a similar content to those identified as possible errors in EC-PC (e.g., Brief-Cope, Diabetes Self-Management Questionnaire).

EC-PC Cronbach’s alpha reflects satisfactory intercorrelations between items of the domains composing the EC-PC, except for domain 3-Self-management that was just under the limit of the acceptable 0.70 threshold value (Table 2). However, qualitative studies^5–7^ showed the suitability of the items contained into Self-management domain and, therefore, this result could be considered acceptable. Item-total correlation coefficients were over the minimal standard threshold, except for the items 12 and 13 that showed a low item-total correlation (Table 2). These statistical results differ from previous qualitative studies,^5–7^ which confirmed that both items are intrinsic characteristics of their respective dimensions. Concerning the inter-item correlation, 85% of the items showed adequate coefficient values according the standards,^11,12^ a fact reflected in satisfactory item homogeneity indexes for all domains. To summarize, most of the EC-PC internal consistency parameters were satisfactory as a whole.

Test–retest reliability was satisfactory for the total scale and for the dimensions, except for domain 3-Self-management that reached an ICC marginally <0.60 (Table 2). The circumstances for data collection in the first application and in the retest were different (see Materials and Methods—Test–retest assessment). This methodological difference could have introduced additional variability in the scores stability and, besides other factors not controlled in the study, could have influenced the responses.^13^

The EC-PC total score precision was satisfactory (Table 2). Nonetheless, the results for the dimensions 2, 3, and 4 were minimally out of the proposed standard, suggesting that those domains may be somewhat less sensitive (precise) than expected. SEM is a fairly stable parameter among different samples,^14^ but future studies will have to confirm the precision weakness of these three EC-PC dimensions if the reliability coefficient (derived from the test–retest) is obtained following a different approach than in the present study.

The correlations of EC-PC with DUFSS and Satisfaction with Life as a whole resulted, as it was hypothesized on the basis of the conceptual relationships between their respective constructs, strong, and moderate, respectively. These findings are consistent with the previous studies^6,7^ showing the relationships between social support and satisfaction with the process of living with PD. However, an unexpected weak relationship between EC-PC and SCOPA-PS was found, suggesting that living with PD was quite independent of the psychosocial adjustment to the disease. From a conceptual point of view, this finding has not an easy explanation and, in fact, an at least moderate association was expected. Some potential reasons for such discrepancy could be (1) both concepts really are loosely related; (2) the EC-PC or the SCOPA-PS does not assess what it intends to measure; or (3) the population participating in the present study has peculiarities in these aspects. None of these speculative explanations, however, seems to be convincing. Again, future studies should verify the finding and clarify these points.

On the other hand, the association between EC-PC and the clinician-based assessments was weak or negligible (Table 3), although a moderate or weak relationship was expected. In this context, the different sources of information (patient versus clinician) and the difference between the constructs evaluated by both types of assessments (e.g., physical signs versus disease acceptance) can explain, at least in part, their weak correlation. In addition, intrinsic individual (e.g., personality and beliefs) and environmental (e.g., social networks and social support) characteristics broadly differ among subjects, circumstances able to influence with a wide degree of variability the EC-PC, but not the PD manifestations.

Internal validity for EC-PC dimensions was satisfactory, except for domain 1-Acceptance (Table 3). This result could be explained with previous qualitative studies,^5–7^ showing that Acceptance of disease is always the first process necessary to achieve a positive living with the condition, because only when the person has accepted his/her illness, can move on to other processes. Thereby, according to the quantitative data emerged in this study and based on the previous qualitative studies, Acceptance is considered an internal, illness-independent, process through which the patient recognizes and assumes the reality.

EC-PC demonstrated satisfactory known-groups validity, yielding significantly different scores between patients with different PD severity levels based on HY and CISI-PD (Table 4). No significant differences were found in regard to the PGIS severity levels due to the closeness of EC-PC scores for mild and moderate levels, although both were higher (better) than for the severe group, as observed with HY and CISI-PD. These results are congruent with the previous studies carried out in PD,^6,7^ showing that patients in early stages of the disease present a better degree of living with PD (positive living) than patients in more advanced stages (negative living).

Main limitations of the study are: (1) the study was not performed on a population basis but in a specialized setting; (2) there are not data on patients with cognitive deterioration, although eight patients (2.5%) meeting all criteria for inclusion were qualified as with cognitive impairment according to the CISI-PD item 4, which was the last assessment performed by the neurologist in the field work; and (3) modification of the procedure between test (in office) and retest (at home), a circumstantial change probably adding spurious variability.

In conclusion, this first validation study of the EC-PC showed that the scale feasibility and acceptability are satisfactory, whereas presents some weaknesses in reliability and precision parameters, although they do not affect the scale total score. As a whole, the analyzed modalities of validity were deemed satisfactory. Additional adaptation and validation studies are needed, in other settings and populations, to identify the shared components of the EC-PC for other chronic conditions and verify its psychometric properties.

## Materials and methods

### Design

This was an open, international, cross-sectional (one point-in-time evaluation, with retest) study.

### Patients

The sample was composed of consecutive PD outpatients from participant centers. Patients were assessed in the period from January to June 2015. Inclusion criteria were: (1) patients with diagnosis of PD by a neurologist according to the international recognized diagnostic criteria;^4^ (2) native Spanish-speaking patients from five countries (Argentina, Cuba, Ecuador, Mexico, and Spain); (3) able to read and understand properly questionnaires; and (4) able to provide informed consent. Exclusion criteria were: (1) parkinsonism other than PD; (2) concomitant severe systemic condition; (3) cognitive deterioration previously and formally diagnosed by the neurologist; (4) acute disorder or injury, pharmacological effect (e.g., dopamine antagonists), or sensorial deficit (e.g., blindness) potentially distorting the assessment; and (5) refusal to participate or patients who did not meet all inclusion criteria.

### Assessments

In addition to sociodemographic and PD historic data, the following measures were used:

HY staging,^15^ a five-level classification universally used to provide a global estimate of PD progression.

Scales for Outcomes in Parkinson’s Disease-Motor (SCOPA-Motor).^16,17^ An assessment including 21 items in three sections: Examination, Activities of daily living, and Motor complications. Each item scores from 0 (normal) to 3 (severe) and the total scale score is 0–75.

The Clinical Impression of Severity Index-PD (CISI-PD)^18,19^ evaluates the global impression of PD severity in four areas: Motor signs; Disability; Motor complications; and Cognitive status. Items are rated from 0 (normal) to 6 (very severe) and the total score runs from 0 to 24.

#### Non-Motor Symptoms Scale

Assessment for the burden of non-motor symptoms in PD.^20^^,^^21^ It includes 30 items in nine domains. Each item is scored for frequency and severity, from 0 (no present) to 12 (maximum frequency and severity). Total scores for the domains and the whole scale can be obtained by the sum of the corresponding items scores.

The SCOPA-PS^9,22^ is an 11-item questionnaire assessing psychosocial functioning during the preceding month. Items are scored from 0 (not at all) to 3 (very much), with higher scores denoting greater difficulty. The summary index is obtained by the sum of item scores transformed into percentage values.

DUFSS^23,24^ is an 11-item scale for assessing diverse dimensions of social support including confidant, affective, and instrumental support. Scores range from 11 (lowest level “much less than I would like”) to 55 (highest level “as much as I like”).

#### Satisfaction with Life Scale

The version used in the present study is a six-item scale to measure the degree of Satisfaction with Life as a whole (item 1) and in regard to five areas: physical, psychological well-being, social relations, leisure, and financial situation.^25^ Each item scores from 0 (unsatisfied) to 10 (totally satisfied).

#### Patient-based Global Impression of Severity Scale

An adaptation of the clinical global impression of severity ^26^ for patients self-application.^27^ It is rated on a six-point Likert-type scale ranging 0 (not ill at all) to 5 (extremely ill).

The EC-PC is a self-reported scale to evaluate how the patient is living with a chronic illness as PD.^10^ For the validation study, a 27-item version was used. However, interim testing of the internal consistency showed poor performance of one item and it was removed. Consequently, the final version of EC-PC is a 26-item scale with five domains: 1-Acceptance (4 items); 2-Coping (7 items); 3-Self-management (4 items); 4-Integration (5 items); and 5-Adjustment (6 items). Items are scored on a five-point Likert scale ranging from 0 (never/nothing) to 4 (always/a lot), except for domain 1-Acceptance that ranges upside down (4: never/nothing; 0: always/a lot). Total score ranges from 0 (negative living with PD) to 104 (positive living with PD).

### Test–retest assessment

For test–retest analysis, the EC-PC was applied a second time to a consecutive subset of stable patients as per the PGIS (test: in office; retest: at home, post mail). A minimum sample of 50 subjects and a time span of 7–10 days for the retest was planned.

### Ethical aspects

The study was approved by the Ethics Committee of the University of Navarre and all participating centers. Patients gave their signed consent to participate after receiving the pertinent information.

### Data analysis

Descriptive statistics (central tendency measures, proportions) were used as needed. Main data were ordinal or did not fit normal distribution; therefore, non-parametric statistics were used. A minimal sample of 10 patients per EC-PC item and 60 per country (*n*=300) was proposed. The following psychometric attributes were tested:

#### Feasibility and acceptability

Quality of data was considered satisfactory if 95% of the data were computable. The limit for missing data was <5%.^11^ Floor and ceiling effect were deemed acceptable if they were <15% ^28^ and the skewness was expected between −1 and +1.^29^

Scaling assumptions were checked attending the range of means and s.d. distribution (roughly equivalent).^11,30^ Also, multitrait-scaling analysis was carried out to check out the appropriate location of items in their correspondent dimensions.^31^

Internal consistency was tested by Cronbach’s alpha coefficient (criterion value >0.70),^32^ item-total correlation (corrected for overlap; criterion value, *r*_s_⩾0.30),^29,33^ inter-item correlation (criterion value, *r*⩾0.20 and ⩽0.75),^11,12^ and item homogeneity (criterion value >0.30).^34^

Reproducibility (test–retest) was determined using weighted kappa (with quadratic weights) for items (standard, >0.41 moderate)^35^ and intraclass correlation coefficient (one way, random effect; ICC) for domains and total score. Values ⩾0.60 were considered acceptable.^31,36^

Precision for each EC-PC domain and total score was estimated by means of the standard error of measurement (s.e.m.=s.d.×√[1−*r_xx_*]),^32^ where *r_xx_* was the reliability coefficient, ICC. A s.e.m. value <½ s.d. was used as criterion of acceptable precision.^37^

#### Construct validity:

For convergent validity, a moderate (*r*_s_⩾0.35–0.50) or strong relationship (*r*_s_>0.50)^38–40^ was hypothesized between EC-PC and DUFSS, SCOPA-PS, and Satisfaction Scale, and a weak/moderate association between EC-PC and the clinical, rater-based PD assessments in the study. Spearman rank correlation coefficients were obtained to this purpose. Internal validity, defined as the intercorrelations between the EC-PC dimensions (standard, *r*_s_=0.30–0.70)^29,33^ and known-groups validity for disease stage (HY); disease severity levels (CISI-PD); and PGIS scores were determined. Mann–Whitney and Kruskal–Wallis tests were used for groups comparison.

Stata 13 (Stata Corp., College Station, TX, USA) was used for data analysis.

## Figures and Tables

**Table 1:** Summary of the measures applied in the study

	*Mean*	*s.d.*	*Median*	*Observed range*	*Theoretical range*
SCOPA-Motor	22.34	11.26	22	1–68	0–75
CISI-PD	7.61	4.00	7	0–24	0–24
Non-Motor Symptoms Scale	60.36	46.76	51	0–243	0–360
SCOPA- Psychosocial	9.71	6.49	8.5	0–29	0–33
DUFSS	27.66	10.00	28	1–44	1–55
Satisfaction with Life (whole)	6.75	1.96	7	0–10	0–10
					
*EC-PC*					
Domain 1. Acceptance	7.90	4.68	8	0–16	0–16
Domain 2. Coping	17.27	6.79	17	0–28	0–28
Domain 3. Self-management	10.35	3.77	10	0–16	0–16
Domain 4. Integration	13.52	4.70	14	0–20	0–20
Domain 5. Adjustment	13.41	6.02	13	0–24	0–24
EC-PC total score	62.45	18.57	62	22–104	0–104

Abbreviations: CISI-PD, Clinical Impression of Severity Index-PD; DUFSS, Duke-UNC Functional Social Support Questionnaire; EC-PC, Living with Chronic illness-PD Scale; PGIS, Patient-based Global Impression of Severity Scale; SCOPA-Motor, Scales for Outcomes in Parkinson’s Disease-Motor; SCOPA-Psychosocial, Scales for Outcomes in Parkinson’s Disease-Psychosocial.

**Table 2:** Feasibility/acceptability, reliability, and precision of the EC-PC

	*EC-PC*				
	*Domain 1. Acceptance*	*Domain 2. Coping*	*Domain 3. Self-management*	*Domain. 4 Integration*	*Domain 5. Adjustment*
Data quality (%)	100	100	100	100	100
Floor effect (%)	3.70	0.93	0.31	0.62	0.93
Ceiling effect (%)	7.41	4.63	12.96	8.02	7.41
Skewness	0.16	−0.29	−0.21	−0.82	0.05
Cronbach’s alpha	0.88	0.86	0.68	0.82	0.79
Item-total correlation	0.64–0.80	0.25–0.80	0.21–0.64	0.42–0.77	0.38–0.76
Inter-item correlation	0.55–0.76	0.14–0.73	0.19–0.69	0.25–0.63	0.16–0.70
Item homogeneity	0.65	0.48	0.42	0.47	0.40
Reproducibility^a^	0.85	0.61	0.57	0.60	0.81
Precision (s.e.m.) (1/2 s.d.)	1.51 (1.96)	2.83 (2.27)	1.70 (1.30)	2.31 (1.83)	2.98 (3.42)

Abbreviation: EC-PC, Living with Chronic Illness Scale.

a Intraclass correlation coefficient.

**Table 3:** Convergent validity and internal validity of EC-PC

	*EC-PC*					
	*Domain 1. Acceptance*	*Domain 2. Coping*	*Domain 3. Self-management*	*Domain 4. Integration*	*Domain 5. Adjustment*	*Total score*
*Convergent validity*						
Hoehn and Yahr staging	−0.17	−0.05	−0.02	−0.13	−0.02	−0.09
CISI-PD	−0.25	−0.07	−0.03	−0.14	−0.15	−0.16
SCOPA-Motor	−0.27	−0.01	0.07	−0.03	−0.14	−0.08
Non-Motor Symptoms Scale	−0.36	−0.23	−0.18	−0.24	−0.19	−0.32
SCOPA-Psychosocial	−0.40	−0.09	−0.08	−0.23	−0.24	−0.25
DUFSS	0.12	0.61	0.57	0.57	0.36	0.61
Satisfaction—With Life as a whole	0.43	0.29	0.28	0.33	0.48	0.46
With physical health	0.32	0.15	0.15	0.26	0.27	0.28
With psychological well-being	0.40	0.22	0.21	0.26	0.34	0.35
With social relations	0.41	0.24	0.23	0.30	0.34	0.38
With leisure	0.40	0.29	0.26	0.36	0.41	0.43
With financial situation	0.38	0.05	0.05	0.10	0.24	0.18
						
*Internal validity*						
Domain 2. Coping	−0.00	—	—	—	—	—
Domain 3. Self-management	0.01	0.78	—	—	—	—
Domain 4. Integration	−0.04	0.71	0.73	—	—	—
Domain 5. Adjustment	0.23	0.54	0.47	0.46	—	—

Abbreviations: CISI-PD, Clinical Impression of Severity Index-PD; DUFSS, Duke-UNC Functional Social Support Questionnaire; EC-PC, Living with Chronic illness-PD Scale; SCOPA-Motor, The Scales for Outcomes in Parkinson’s Disease-Motor; SCOPA-Psychosocial, Scales for Outcomes in Parkinson’s Disease-Psychosocial.

**Table 4:** Known-groups validity

*Categories*	*EC-PC total*	P *value*^a^
*HY-based severity levels*^b^		
Mild	14.15±4.80	<0.001
Moderate	13.73±4.42	
Severe	9.42±3.31	
		
*CISI-PD severity levels*^c^		
Mild	64.15±18.58	<0.001
Moderate	61.99±18.50	
Severe	46.73±10.35	
		
*PGIS-based severity levels*^d^		
Mild	63.28±19.33	NS
Moderate	64.94±17.15	
Severe	47.03±13.37	

Abbreviations: CISI-PD, Clinical Impression of Severity Index for Parkinson’s Disease; EC-PC, Living with Chronic illness-PD Scale; HY, Hoehn and Yahr staging; NS, nonsignificant; PGIS, Patient-Based Global Impression of Severity Scale.

a Kruskal–Wallis test.

b HY-based severity levels: mild, stages 1 and 2; moderate, stage 3; severe, stages 4 and 5.

c CISI-PD severity levels: mild 1–7 points; moderate 8–14 points; severe 15 or more points.

d PGIS-based severity levels: mild 0–2 points; moderate 3 points; severe 4 or more points.
